# The Principles and Practice of Dentistry

**Published:** 1885-08

**Authors:** 


**The Principles and Practice of Dentistry.** Including Anatomy, Physiology, Pathology, Therapeutics. Dental Surgery and Mechanism. By CHAPIN A. HARRIS, M.D., D.D.S. Eleventh edition. Revised and edited by Ferdinand J. S. GORGAS, A.M., M.D., D.D.S. With two full-sized plates and 744 illustrations. Cloth, $6.50; sheep, $7.50. Philadelphia: P. Blakiston, Son & Co., 1012 Walnut street. 1885.

This is a large work of 994 pages, devoted entirely to the principles and practice of Dentistry. It presents the subject to which its pages are devoted, with the latest improvements in this most important department. To the dental surgeon, the work supplies a wealth of information which will be of incalculable aid in his profession. We commend the work to our dental readers, and regard it as a very able exposition of the subject to which its pages are devoted.

